# Oil Spill Detection Performance in a Multitype Polarimetric-Feature Space Using a Polarimetric Synthetic Aperture Radar: A Comparative Analysis

**DOI:** 10.3390/s26154750

**Published:** 2026-07-26

**Authors:** Guannan Li, Gaohuan Lv, Xiang Wang, Fen Zhao, Xiluo Teng

**Affiliations:** 1 Ulsan Ship and Ocean College, Ludong University, Yantai 264025, China; liguannan@ldu.edu.cn (G.L.); lvgh@ldu.edu.cn (G.L.); 2 Ocean Remote Sensing Division, National Marine Environmental Monitoring Center, Dalian 116026, China; xwang@nmemc.org.cn; 3 School of Resources and Environmental Engineering, Ludong University, Yantai 264025, China; 4 Faculty of Engineering and Information Technology, The University of Melbourne, Parkville, VIC 3010, Australia; xiluot@student.unimelb.edu.au

**Keywords:** marine oil spills, polarimetric synthetic aperture radar, comparative analysis, multitype feature space

## Abstract

**Highlights:**

**What are the main findings?**

- In experiments involving different incident angles, polarimetric features related to the scattering mechanism and channel correlation are useful in identifying different oil slick types.
- Features combining the scattering entropy *H* and modified anisotropy *A*_12_ can be used to reliably identify oil slick types at various incident angle conditions.

**What are the implications of the main findings?**

- The results can be used to identify the polarimetric features related to the scattering mechanism and channel correlation, providing reliable information on marine oil spills; additionally, these features are less affected by the incident angle.
- These polarimetric features by combining the advantageous polarimetric features corresponding different incidence angle conditions can be incorporated in oil spill identification and monitoring to improve efficiency.

**Abstract:**

Marine oil spills severely threaten marine ecosystems, the coastal economy, and marine engineering structures. Because it enables all-weather and all-time acquisition of rich target information, fully polarimetric synthetic aperture radar (FP SAR) is widely used for monitoring marine oil spills. However, the differences in the scattering characteristics among oil types can cause variability in the information contained in the features extracted using FP SAR. Herein, RADARSAT-2 images obtained from a rare oil-on-water experiment conducted in the Norwegian North Sea were used to compare the distribution differences in polarimetric features based on the oil slick type and incident angle. Results showed that the incident angle exerted some influence on polarimetric features and the detection performance for oil spills with a low oil–water contrast, particularly at large incident angles. The polarimetric features related to scattering mechanisms exhibited good robustness and effectiveness across various incident angles. The polarimetric feature that combines the scattering entropy *H* and modified anisotropy *A*_12_ exhibited strong overall performance and high suitability for extracting information on oil spills at different incident angles. This study demonstrates that incorporating appropriate polarimetric features according to the incident angle enables the identification of different oil slick types and facilitates oil spill detection and monitoring.

## 1. Introduction

Marine oil spills are primarily caused by (i) human activities, such as leakage from drilling platforms or ship collisions, and (ii) natural seepage from the seabed [1]. They have received considerable public attention because of the damage they cause to the ecological environment, fisheries, and other societal interests [2,3]. The development of satellite-based remote-sensing technology has substantially contributed to the large-scale monitoring of marine environments and to the implementation of disaster-warning systems [4]. Polarimetric synthetic aperture radar (SAR) has the advantages of all-weather and all-time microwave detection, which can effectively reduce the constraints posed by weather conditions; additionally, it can obtain rich target information, including intensity and phase information. These advantages are conducive for the early warning and near-real-time monitoring of oil spills as well as for post-disaster emergency response efforts [3,5]. Although fully polarimetric (FP) SAR needs to balance radar antenna technology, system energy consumption, and data storage and download, its bandwidth is only half that of dual-polarization SAR [6]. However, it can record and obtain sufficient intensity and phase information for each distinguishable pixel in an image, thus providing more physically reliable and comprehensive results for identifying and extracting marine targets compared with single- and dual-polarization SAR.

Many studies have focused on comparing different features that can be extracted from FP SAR to obtain accurate information on oil spills [7,8,9]. Several studies have classified polarimetric features to evaluate their oil spill recognition performance of different feature types [9,10] with respect to various aspects, including oil formation mechanisms [6], oil slick thicknesses [7], oil slick types [11], and oil spill scenarios [12]. Some features are affected by the system configuration and observation conditions, possibly leading to differences in depicting the target characteristics [13]. Furthermore, the characteristics of polarimetric features differ according to the wind speed and incident angle [9]. The information contained within a given polarimetric feature depends not only on the incident angle but also on the polarimetric mode [14]. Studies have fully demonstrated the advantages of polarimetric SAR in oil spill detection. Quantitative comparisons of the differences in oil spill information contained within different polarimetric features have been reported in some studies [15,16,17,18]. Furthermore, comparative analyses of different polarimetric features and detection conditions have been conducted to investigate the effects of factors such as the wind direction, wind speed, incident angle, and local noise on such features [9,13,19]. However, a comprehensive evaluation of the information extracted from polarimetric features for different types of oil slick (i.e., mineral and plant oil) has not been reported. Therefore, further research is needed to investigate the effect of the incident angle on different polarimetric features. A deeper understanding of various polarimetric features and the mechanism through which environmental conditions influence them is required to identify features that exhibit a low dependence on environmental factors and reliable information for identifying marine oil spills.

Based on the aforementioned background, in this study, a comparative analysis was conducted on FP SAR data obtained from an oil spill experiment involving different incident angles; the objective was to clarify the reliability of different polarimetric features in providing information on different oil slick types. The primary contributions of this study are as follows:

1. The ability of various types of polarimetric features employed in the detection of marine oil spills to provide information on different oil slick types at different incident angles is comprehensively evaluated.
2. The potential effect of the incident angle on polarimetric features is analyzed, and the optimal features for different incident angles are identified.

Notably, this study conducted a rare marine oil spill experiment to obtain different incident angle datasets under the same oil spill scene conditions. Despite effectively avoiding errors caused by different sensors and study areas, the comparisons and analyses conducted using a limited dataset may lead to different influencing factors and results in other scenarios and observation conditions. This study is anticipated to guide the performance assessment of polarimetric features for detecting oil spills.

## 2. Materials and Methods

### 2.1. Experimental Data

**Figure 1:**
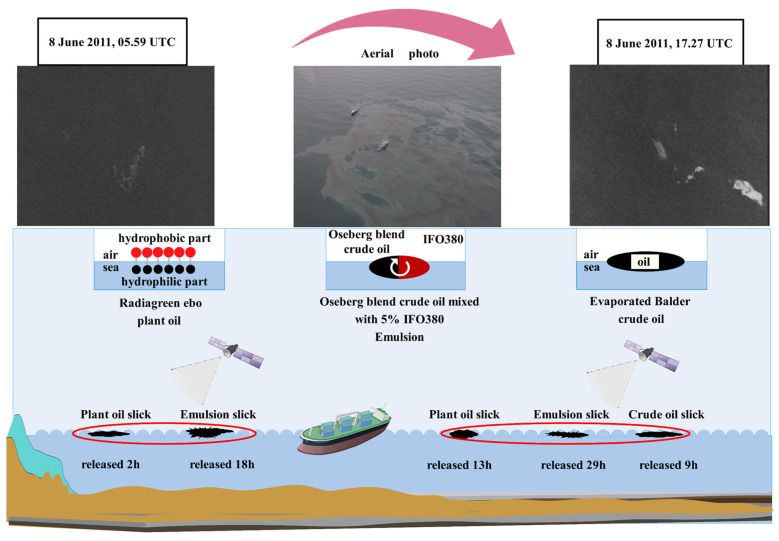
Formation of different oil slick types in experiment.

**Figure 2:**
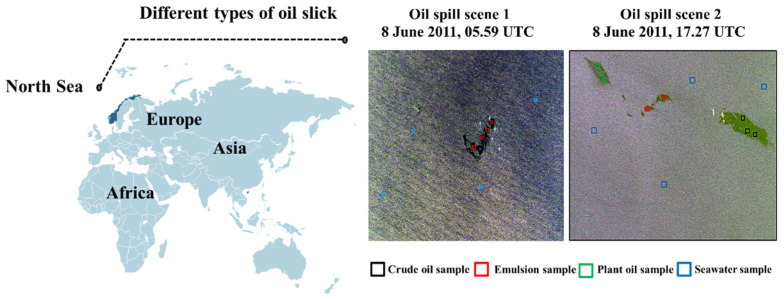
Geographic location of RADARSAT-2 images of Scenes 1 and 2 (black: crude oil, red: emulsified oil, green: plant oil, blue: seawater).

The image data were obtained in the manufactured oil-on-water experiment (from 6 to 9 June 2011) in the North Sea (59°59′ N, 2°27′ E), conducted by the Norwegian Clean Seas Association for Operating Companies (NOFO), which was observed by the Earth observation satellite RADARSAT-2, equipped with a C-band SAR system [20]. Their experimental data provided a rare opportunity to analyze the polarimetric scattering characteristics of different oil slick types at different incident angles. Two oil slick types were considered: a mineral oil slick and a plant oil slick. The crude oil and emulsified oil (Oseberg blend crude oil mixed with 5% IFO3801), and the plant oil was a 2-ethylhexyl oleate, which was used to simulate a natural monomolecular biogenic oil slick. The data acquisition time interval for the two scenarios was 11 h. Figure 1 shows the experimental process and investigation scenarios. Figure 2 shows the RADARSAT-2 images of Scenes 1 and 2 with the crude oil, emulsified oil, plant oil, and seawater color-coded as black, red, green, and blue boxes, respectively. Table 1 provides detailed information about the RADARSAT-2 data used to characterize the different oil slick types. Two unique scenes were considered: Scene 1 occurred at 5:59 UTC on 8 June 2011 and corresponded to large incident angles of 46.1–47.3°. Scene 2 occurred at 17:27 UTC on 8 June 2011 and corresponded to small incident angles of 34.5–36.1°. A wind speed of 1.6–3.3 m/s was recorded by the ships participating in the experiment. All subsequent analyses were conducted using subsets of the entire dataset.

### 2.2. Theory of the FP SAR System

The FP SAR system transmits and receives polarization channels in four linear combinations to obtain rich information (including intensity and phase information) about the target [3,5,11,21]. Numerous studies have investigated the effectiveness and superiority of the FP SAR system in the detection of marine oil spills, such as polarimetric feature extraction and oil spill recognition algorithms [1,11,12,17,21,22,23].

For the scattering matrix of a single/pure target, under the reciprocity assumption *S_HV_* = *S_VH_*, the scattering matrix in the FP mode with its three-dimensional Lexicographic-basis vector *k_L_* and Pauli-basis vector *k_P_* is given as [5]

(1) $$S=\left[\begin{array}{cc} S_{HH} & S_{HV}\\ S_{VH} & S_{VV} \end{array}\right]\Rightarrow \left\{\begin{array}{c} k_{L}={\left[\begin{array}{ccc} S_{HH} & \sqrt{2}S_{VH} & S_{VV} \end{array}\right]}^{T}\\ k_{P}={\left[S_{HH}+S_{VV},S_{HH}-S_{VV},2S_{HV}\right]}^{T}/\sqrt{2} \end{array}\right.$$

where *C*_3_ and *T*_3_ are given as [5,20,23]

(2) $$C_{3}=\langle k_{L}k_{L}^{*T}\rangle =\left[\begin{array}{ccc} \langle {\left|S_{HH}\right|}^{2}\rangle & \sqrt{2}\langle S_{HH}S_{HV}^{*}\rangle & \langle S_{HH}S_{VV}^{*}\rangle \\ \sqrt{2}\langle S_{HV}S_{HH}^{*}\rangle & 2\langle {\left|S_{HV}\right|}^{2}\rangle & \sqrt{2}\langle S_{HV}S_{VV}^{*}\rangle \\ \langle S_{VV}S_{HH}^{*}\rangle & \sqrt{2}\langle S_{VV}S_{HV}^{*}\rangle & \langle {\left|S_{VV}\right|}^{2}\rangle \end{array}\right]$$

(3) $$T_{3}=\langle k_{P}k_{P}^{*T}\rangle =\frac{1}{2}\left[\begin{array}{ccc} \langle {\left|S_{HH}+S_{VV}\right|}^{2}\rangle & \langle \left(S_{HH}+S_{VV}\right){\left(S_{HH}-S_{VV}\right)}^{*}\rangle & 2\langle \left(S_{HH}+S_{VV}\right)S_{HV}^{*}\rangle \\ \langle \left(S_{HH}-S_{VV}\right){\left(S_{HH}+S_{VV}\right)}^{*}\rangle & \langle {\left|S_{HH}-S_{VV}\right|}^{2}\rangle & 2\langle \left(S_{HH}-S_{VV}\right)S_{HV}^{*}\rangle \\ 2\langle S_{HV}{\left(S_{HH}+S_{VV}\right)}^{*}\rangle & 2\langle S_{HV}{\left(S_{HH}-S_{VV}\right)}^{*}\rangle & 4\langle {\left|S_{HV}\right|}^{2}\rangle \end{array}\right]$$

The oil spill detect ability in a SAR image is affected by the system background noise to a certain extent; this is commonly referred to as noise equivalent sigma zero (NESZ). This includes aspects such as the description of oil spill characteristics and the expression of target polarimetric information. NESZ can be regarded as a baseline for evaluating the distribution of normalized radar cross-section (NRCS) signals from various oil spill targets and can be used to determine the degree of influence noise exerts on a signal based on its relative distribution relationship. Based on the differences in NESZ, the sensors receive different scattering information from the same target. For RADARSAT-2, ranges [−27.5, −43] dB [19,20]. The NRCS data below the NESZ baseline are regarded as corrupted by noise. Therefore, the signal-to-noise level analysis of a given instrument is an important and necessary consideration in target data analysis. Comparing the relative distributions of marine targets (e.g., oil spills and seawater) and noise limits is useful for extracting polarimetric information, feature application, imaging condition selection, and algorithm construction under different incident angle conditions and oil spill scenarios. In this study, the differences between targets and corresponding NESZ baselines were compared and analyzed. The visualization of the relative differences among various oil spills, background seawater, and the NESZ baseline, this is different from the y-axis display in traditional signal analysis diagrams; however, the overall distribution trend is the same.

Studies have proposed or improved many polarimetric features for identifying marine oil spills. These features are classified into three main categories [9]: scattering mechanism-, channel correlation-, and scattering energy-based features. Table 2 summarizes representative features of each type that are commonly used in oil spill detection.

### 2.3. Quantitative Evaluation Indicators

#### 2.3.1. Michelson Contrast (MC)

MC, which was initially proposed to quantify the difference in contrast between different targets in an image, measures the relative feature differences between two target samples *i* and *j* [20,27]. In this study, it was applied to oil spills to assess the differences between targets in a one-dimensional feature space. Currently, MC is a common indicator of the degree of separation between oil spill targets. It is defined as follows:

(4) $$MC=\frac{\left|I_{i}-I_{j}\right|}{I_{i}+I_{j}}$$

where *I_i_* and *I_j_* represent the average values of two sample sets of the targets in a certain polarimetric feature space. The range of MC is 0–1.

#### 2.3.2. Overlap Ratio (OR)

We separately extracted the same number of samples of an oil slick and background seawater in each one-dimensional feature space employed in this study, counted the number of overlapping samples between pairwise targets, and calculated the proportion of overlapping samples [14]. This allowed us to quantitatively assess the intra-class confusion degree in the one-dimensional feature space. The OR is defined as follows:

(5) $$OR=\frac{m_{i\&j}}{M}$$

where $m_{i\&j}$ is the statistical quantity of the overlapping samples of two targets *i* and *j* and *M* represents the total number of samples. In this study, the same number of samples was selected for all targets to facilitate pairwise comparisons under the same evaluation criterion.

#### 2.3.3. Variable Importance Analysis

**Figure 3:**
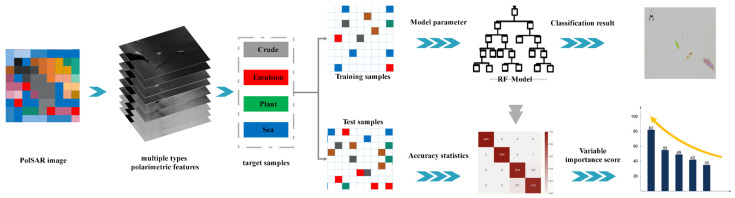
Implementation process of Image-RF under the EnMAP-box platform.

The six polarimetric features listed in Table 2 are used as inputs in the random forest (RF) algorithm to detect oil spills. RF is a suitable classifier because it integrates multiple classification and regression trees. Additionally, it can randomly select sample subsets and feature subsets to effectively suppress the overfitting caused by samples and noise. Furthermore, it can be used to quantitatively evaluate the variable importance of input features, which is useful for analyzing advantageous polarimetric features to detect oil spills. In this study, the RF was implemented using EnMAP-Box, which is a toolbox widely used for remote-sensing image processing and analysis. Figure 3 shows the implementation process of RF algorithm. The following steps were implemented:

1. A stack of multiple types of polarimetric features was generated with six feature variables, which cover the aforementioned scattering mechanisms, channel correlations, and scattering energy type features.
2. A subset was randomly selected from each type of target area (i.e., crude oil, emulsified oil, plant oil, and seawater). For each category, 10,000 pixel points were randomly selected (in Figure 2). However, for plant oil, 5000 pixel points were chosen due to its relatively small area. Among them, 70% of these were used as the training sample set, and the remaining 30% were used as the test sample set.
3. The RF was used to identify oil spills in a RADARSAT-2 image using the selected features as inputs; the contributions of each feature to the classification results were calculated. The number of decision tree nodes *N* is 100. The mean decrease Gini index is used as the indicator to measure the importance of the feature variables.

## 3. Results

### 3.1. NESZ Analysis

**Figure 4:**
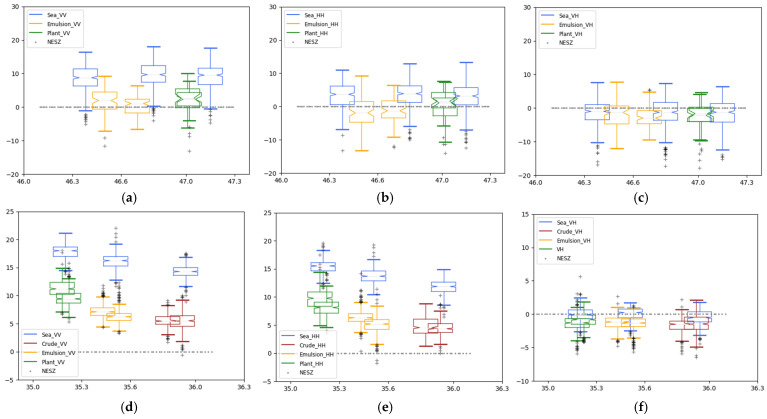
Backscatter signals of different targets relative to the NESZ (*y* = 0) as a function of the incident angle: (**a**) VV channel, (**b**) HH channel, and (**c**) VH channel for Scene 1; (**d**) VV channel, (**e**) HH channel, and (**f**) VH channel for Scene 2. The colors correspond to the boxes shown in Figure 2.

A signal-to-noise analysis was conducted for Scenes 1 and 2. Figure 4 shows the difference between the backscatter signal of various targets and the NESZ baseline (*y* = 0) as a function of the incident angle. In both scenes, the co-polarization channel VV had the largest backscatter signal and exhibited the largest difference with the corresponding NESZ baseline. The co-polarization channel HH was closer to the NESZ baseline. The backscatter signal of the vertical transmit-horizontal receive (VH) cross-polarization channel was mostly below the NESZ baseline, which indicated that this signal was the most affected by noise. Among the oil spill targets, the backscatter signal of the mineral oil slick was the closest to the NESZ baseline followed by the backscatter signal of the plant oil slick; the backscatter signal of seawater exhibited the greatest difference with the NESZ baseline. Between the two scenes, the backscatter signals of the oil slick and seawater in the VV and HH channels were closer to the NESZ baseline in Scene 1 (large incident angles) than those in Scene 2 (small incident angles). In Scene 1, the backscatter signals of the mineral oil slick, plant oil slick, and seawater in the VV channel ranged from −7.1 to 9.3 dB, −6.1 to 9.6 dB, and −2.4 to 18.07 dB, respectively. In Scene 2, the corresponding backscatter signals ranged from 1.6 to 11.3 dB, 6.6 to 15.6 dB, and 13.8 to 21.16 dB, respectively. Regarding the VH channel, the backscatter signals of all targets fluctuated around the NESZ baseline in both scenes. Furthermore, we also compared the statistical indicators of the signal-to-noise differences between different targets and NESZ in the two scenes (shown in Table 3). The results indicated that the backscatter signal of the targets in the HH channel decreased faster than that in the VV channel, thus being more affected by background noise, which is also consistent with the result in [20]. Additionally, the standard deviation of the signal distribution of the targets in the VV channel was overall lower than that in the HH channel in both scenes. The signal of each target in the large incidence angle scenario was closer to NESZ than that in the small incidence angle scenario, and the signal distribution of the targets in the large incidence angle scenario was more dispersed, which can also be known from the standard deviation values in Table 3. It should be noted that the target signal in VH channel fluctuated around the NESZ baseline for both scenes, which was not listed in the comparison.

### 3.2. Oil Spill Identification Capability Analysis of Multi-Type Polarimetric Features

**Figure 5:**
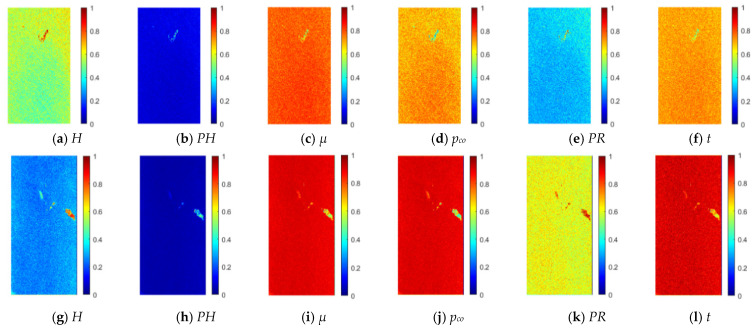
Visualization of the selected polarimetric features in Scene 1 (first row) and Scene 2 (second row).

**Figure 6:**
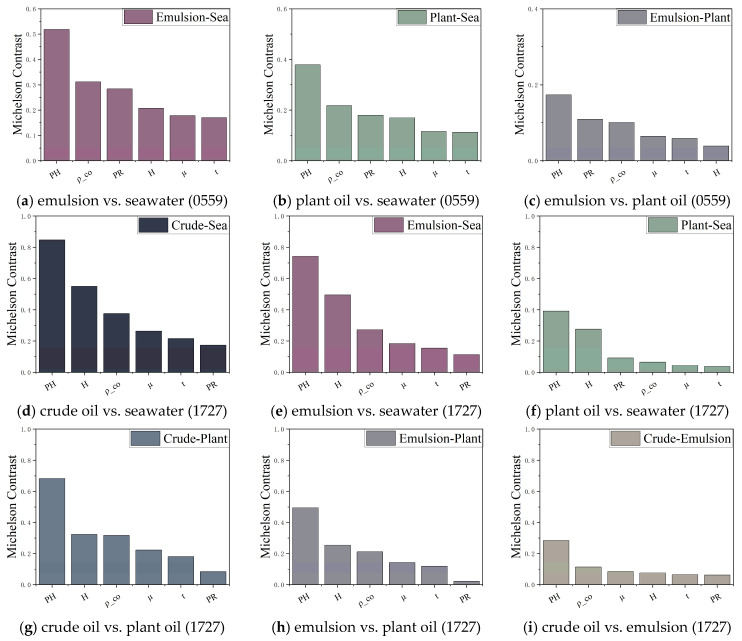
MC results for different polarimetric features. (**a**–**c**) for Scene 1, and (**d**–**i**) Scene 2.

Figure 5 shows the oil spill detection performance of the six selected polarimetric features at different incident angles. Each feature represents the contrast between an oil slick and seawater to some extent. The differences between oil and water characteristics at different incident angles were consistently expressed in different feature spaces. To accurately evaluate the differences in information contained in different types polarimetric features at different incident angles, we compared the MC results of these features for various oil slicks and seawater under two scenarios (Figure 6 and Table 4). Although the overall ranking of the polarimetric features varied depending on the target, in most cases, the trends were consistent. In Scene 1, *PH* and *p_co_* exhibited the best performance. In Scene 2, in most cases (i.e., mineral oil vs. seawater, plant oil vs. seawater, or mineral oil vs. plant oil), *PH* and *H* exhibited the best performance, followed by *p_co_*. These results indicate that the features related to the scattering mechanism exhibit superior oil spill detection performance at different incident angles compared with features related to channel correlation. In most cases, the features related to the scattering energy exhibited slightly lower performance than the other two types of features.

**Figure 7:**
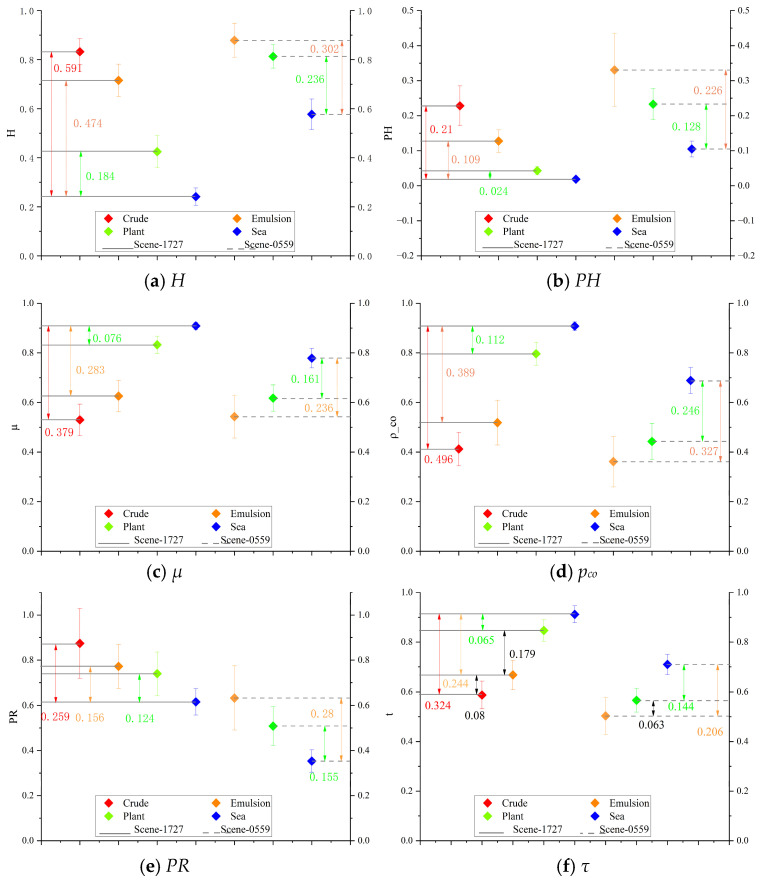
Mean and variance distributions of different target signals for selected features.

In addition to the between-class differences, the distribution of intra-class data determines the ability of a polarimetric feature to distinguish between targets. The mean and standard deviation of the polarimetric features were calculated for different areas of the scenes corresponding to different targets. The results are presented in Table 5 and Figure 7. In most cases, *PH* exhibited the smallest within-class variation, followed by *H*. For small incident angles (Scene 2), the data span corresponding to different oil spill targets was large in various feature spaces. At large incident angles (Scene 1), the dispersion of sample data was large. The MC value of *PH* was the smallest between oil spills and seawater because of the different target distribution over a small range; however, its clustering performance for various targets was high, especially for seawater. This result is validated by the standard deviation of the MC values. The difference between mineral oil and seawater was higher in Scene 2 (small incident angles) than in Scene 1 (large incident angles). Furthermore, the difference between plant oil and seawater was higher in Scene 1 (large incident angles). Among the three types of polarimetric features, those related to the scattering mechanism and channel correlation exhibited large differences among targets. Overall, the results indicated that features related to the scattering mechanism are the most suitable for detecting oil spills because of their large inter-class difference and small intra-class difference.

#### 3.2.1. Comparison of Pairwise Sample Overlap

**Figure 8:**
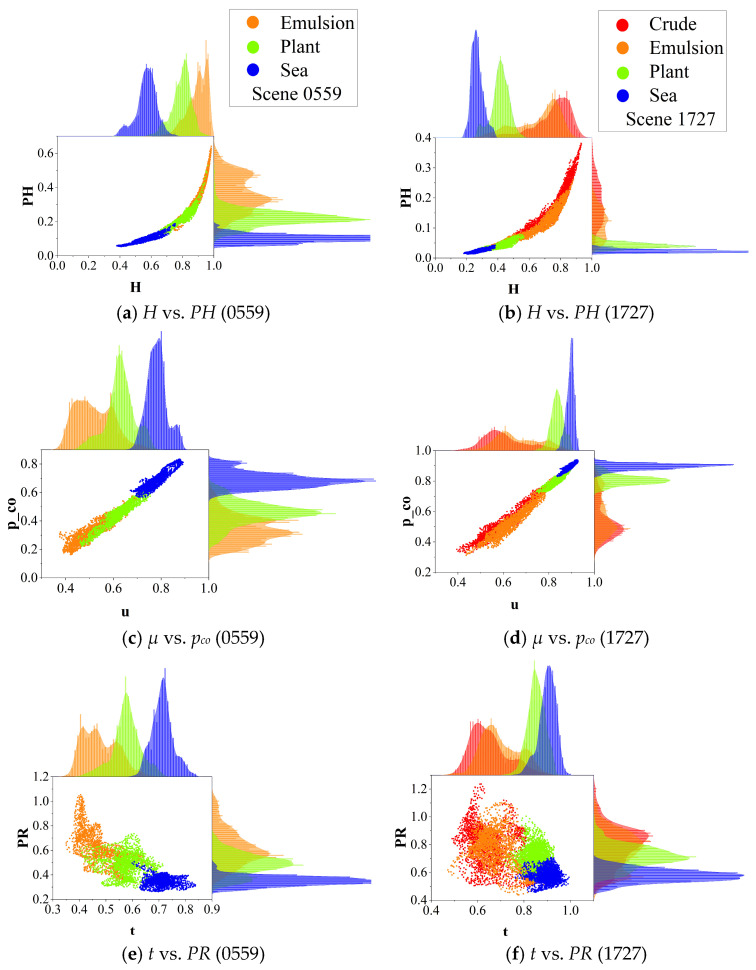
Plot of the probability distribution functions (PDFs) of the polarimetric features in the selected regions.

An effective polarimetric feature capable of separating targets and avoiding confusing targets is essential for detecting oil spills. ORs were calculated for different targets in different polarimetric feature spaces for both scenes (Figure 8 and Table 6). Compared with other features, the features related to the scattering mechanism achieved a better clustering performance for various targets with a large difference in data ranges among targets. The features related to channel correlation also achieved good clustering performance. Both the data differences and the clustering degree of the intra-target data of various types of targets were better. These results agree with the measured mean and variance (Table 5). In both scenes, *PH* and *p_co_* consistently achieved the best results, followed by *H*. Furthermore, the ORs were generally low between mineral oil and seawater and between plant and mineral oil. However, the ORs were high between crude and emulsified oil and between plant oil and seawater; this can be attributed to their similar scattering characteristics.

#### 3.2.2. Ordering Results of Variable Importance

**Figure 9:**
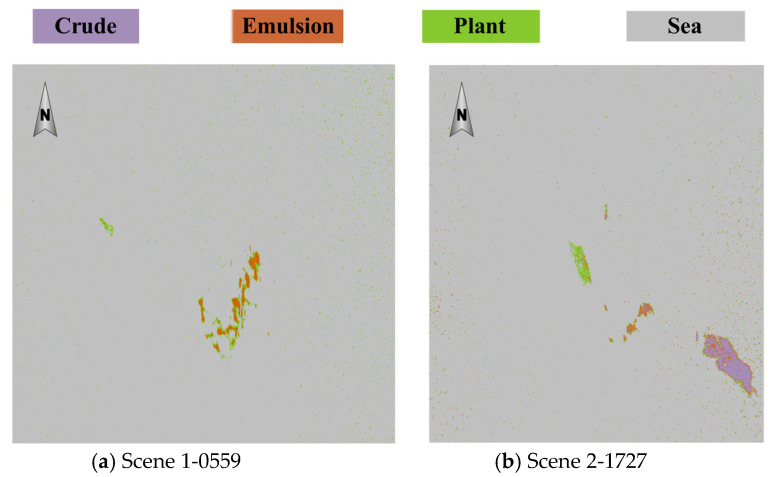
Classification results based on the RF classifier for the study area: (**a**) Scene 1; (**b**) Scene 2.

**Figure 10:**
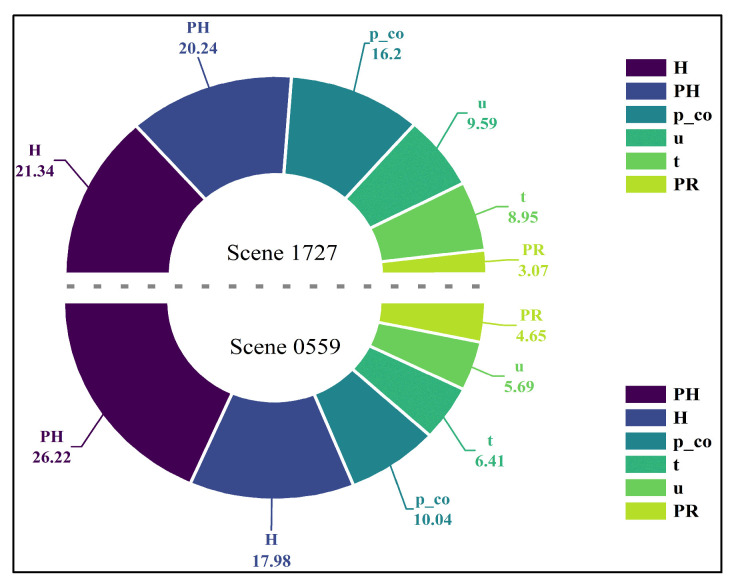
Variable importance of selected polarimetric features in the RF classification results.

Figure 9 and Figure 10 respectively show the oil spill detection results and the ranking of feature importance for the two scenes based on the RF classifier. Good classification accuracy was observed for different types of oil slicks. However, as shown in Table 7, some misclassification occurred. In Scene 2 (small incident angles), the edge samples of crude and emulsified oil were misclassified as plant oil; the classification accuracy was slightly lower for emulsified oil than that for plant oil. In Scene 1 (large incident angles), the classification accuracy was slightly lower for plant oil than that for emulsified oil.

Furthermore, we compared the RF classification results using different feature types as inputs under the same training set/test set (shown in Table 7). This comparison covered three types, two types, and one type of polarimetric feature, respectively. Since the contribution of the scattering energy-based features were relatively low, features based on scattering mechanisms and channel correlations were included in the two types of features. The results showed that the classification results obtained using input features based on scattering mechanisms and channel correlations were very close to the results combining three types. This result also confirmed that the scattering energy-based features contributed relatively less to the classification (shown in Figure 10). Meanwhile, the scattering mechanism-based features contributed more. The classification results were slightly inferior to those of features based on two or three types but still close to the best results.

Figure 10 shows the variable importance of the features used for classifying oil slicks in the two scenes. Overall, the best and second-best performances were achieved by the features related to the scattering mechanism, i.e., *PH* and *H*, respectively, followed by the features related to channel correlation. For Scene 1 (large incident angles), *PH* exhibited the highest variable importance value of 26.22, followed by *H* (17.98) and *p_co_* (10.04). For Scene 2 (small incident angles), *H* exhibited the highest variable importance value of 21.34, followed by *PH* (20.24) and *p_co_* (16.2).

### 3.3. Analysis of the Scattering Mechanisms-Based Feature Combinations (H_A and H_A_12_)

Some studies have combined different types of polarimetric features to improve the oil spill detection performance, increasing both the information difference between an oil slick and the background environment [8,28,29,30], as well as the amount of information available for detection models and algorithms [19]. A study has demonstrated that combining the polarization entropy *H* with the modified anisotropy *A*_12_ achieves superior oil spill detection performance than combining *H* with the conventional anisotropy *A*, particularly regarding the relative thickness of oil slicks [7]. However, the effects of the incident angle on the oil spill detection performance for different feature combinations have not been comprehensively investigated. Therefore, we analyzed the effectiveness of combining *H* and *A*_12_ for detecting oil spills in Scenes 1 and 2.

#### 3.3.1. Construction of Feature Combinations

Equations (1)–(3) can be combined to obtain the coherent matrix of the FP SAR system. The eigenvalues are defined and extracted as follows [5]:

(6) $$\langle T_{3}\rangle =U_{3}\left[\Sigma \right]U_{3}^{-1}=\sum_{i=1}^{i=3}\lambda_{i}T_{i}=\sum_{i=1}^{i=3}\lambda_{i}u_{i}u_{i}^{T*}$$

where *λ_i_* and *u_i_* represent the eigenvalues and eigenvectors, respectively, of the three-dimensional coherent matrix. *A* can be extracted using the two smallest eigenvalues [5]:

(7) $$A=\frac{\lambda_{2}-\lambda_{3}}{\lambda_{2}+\lambda_{3}}$$

*H* and *A* can be combined as follows [30]:

(8) $$H\text{\_}A=\left\{\begin{array}{c} HA\\ H\left(1-A\right)\\ A\left(1-H\right)\\ \left(1-H\right)\left(1-A\right) \end{array}\right.$$

The combined features can be modified by replacing *A* with the improved anisotropy *A*_12_, which is calculated from the difference between the two largest eigenvalues of the coherent matrix [20]. *H* and *A*_12_ can be combined as follows [7]:

(9) $$H\text{\_}A_{12}=\left\{\begin{array}{c} HA_{12}\\ H\left(1-A_{12}\right)\\ A_{12}\left(1-H\right)\\ \left(1-H\right)\left(1-A_{12}\right) \end{array}\right.$$

#### 3.3.2. Comparison of a Combined Feature with Its Subfeatures

**Figure 11:**
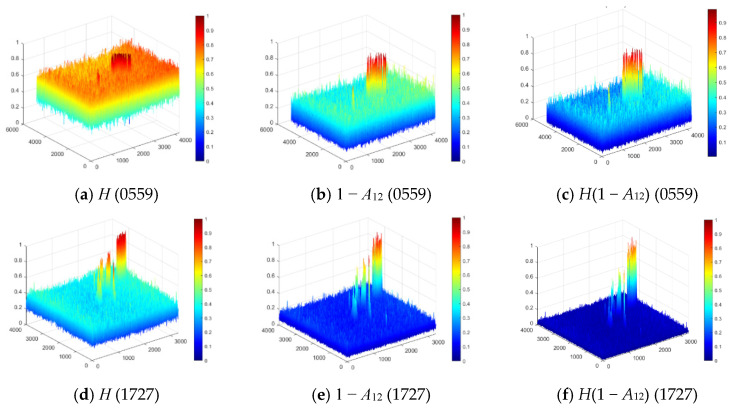
Oil spill detection performance of the combined feature *H*(1 − *A*_12_) and its sub-features: (**a**) *H*, (**b**) 1 − *A*_12_, and (**c**) *H*(1 − *A*_12_) in Scene 1(0559); (**d**) *H* (**e**) 1 − *A*_12_, and (**f**) *H*(1 − *A*_12_) in Scene 2 (1727).

Figure 11 shows the 3D visualization of *H*(1 − *A*_12_) and its subfeatures for Scenes 1 and 2. *H*(1 − *A*_12_) exhibited a larger inter-class difference than its subfeatures, which ensured that the oil spills information was retained while the noise from the seawater was suppressed [7]. The sea surface information was smoother, the sea clutter information peaks were weaker, and the oil–water distinction was stronger. *H*(1 − *A*_12_) exerted a certain damping effect on the plant oil information and performed similarly in both scenes.

Table 8 shows a comparison of the inter-class differences between *H*(1 − *A*_12_) and its sub-features *H* and 1 − *A*_12_ in terms of the feature ratio ($R_{p}$) [23] and MC [21,25]. The feature ratio is calculated as follows:

(10) $$R_{p}=\frac{I_{slick}}{I_{slick\_free}}$$

where *I*_slick_ is the intensity of a given feature within an area covered by an oil slick and *I*_slick_free_ is the intensity of the same feature within an area free of any oil slick. For the feature ratio and MC, *H*(1 − *A*_12_) achieved a superior performance compared with its sub-features *H* and 1 − *A*_12_, especially in distinguishing between mineral oil and seawater. The quantitative results for the feature ratio and MC were generally better in Scene 2 (small incident angles) than those in Scene 1 (large incident angles). *H* represents the randomness of the scattering mechanism of a target signal; *A*_12_ is also related to the scattering mechanism. The visualization results for Scenes 1 and 2 showed that this combined feature is better in representing oil–water differences at small incident angles than at large incident angles.

#### 3.3.3. Comparison Between Feature Combinations H_A and H_A_12_

**Figure 12:**
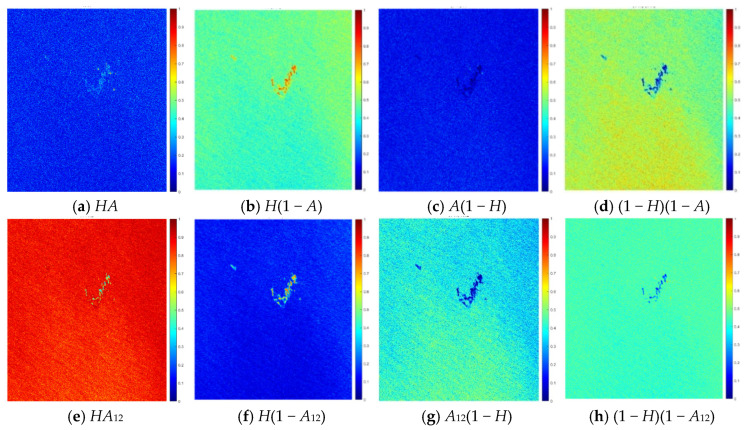
Visualization of *H*_*A* and *H*_*A*_12_ in Scene 1.

**Figure 13:**
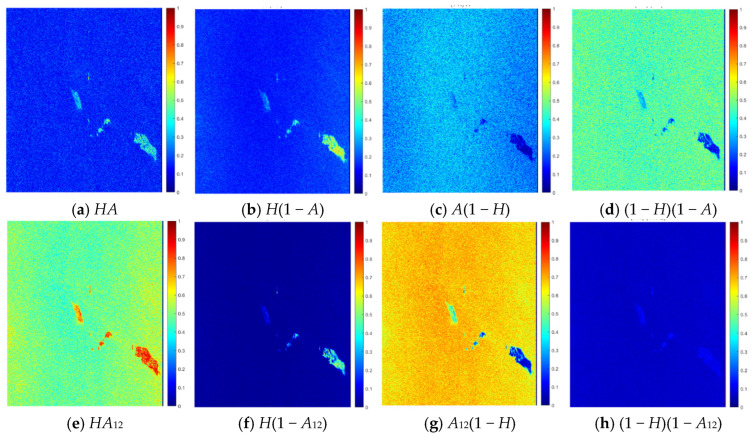
Visualization of *H*_*A* and *H*_*A*_12_ in Scene 2.

Figure 12 and Figure 13 show the visualization results of *H_A* and *H*_*A*_12_ in Scenes 1 and 2, respectively. In general, *H*_*A*_12_ yielded a greater difference between targets than *H_A*, except for (1 − *H*) (1 − *A*_12_). For *H_A* and *H*_*A*_12_, the differences in intensity between oil and water were generally higher in Scene 2 (small incident angles) than those in Scene 1 (large incident angles). Within the two feature sets, *H*(1 − *A*_12_) and *H*(1 − *A*) were most suited for oil spill detection because they enhanced the oil slick signal while suppressing the seawater signal and weak damping features. In addition, *H*(1 − *A*_12_) was more stable and effective and exhibited extremely low intensity for areas free of oil slicks at different incident angles.

**Figure 14:**
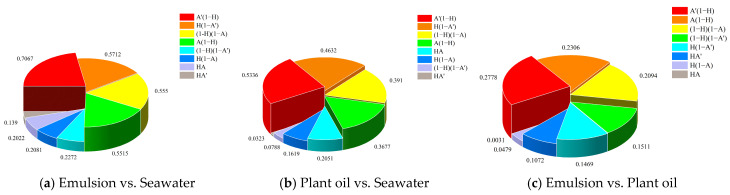
MC ordering results for *H_A* and *H_A*_12_ in Scene 1.

**Figure 15:**
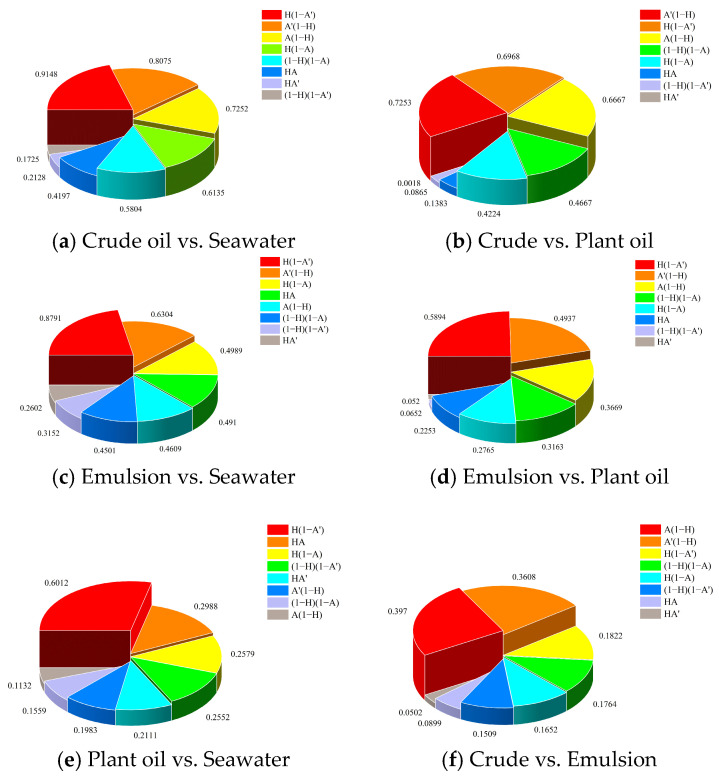
MC ordering results for *H_A* and *H_A*_12_ in Scene 2.

The discrimination performances of *H_A* and *H*_*A*_12_ for different targets were compared for different incident angles. Table 9 summarizes the MC results, and Figure 14 and Figure 15 show the MC ordering results for Scenes 1 and 2, respectively. In Scene 1 (large incident angles), *A*_12_(1 − *H*) produced the best MC results between targets, followed by *H*(1 − *A*_12_). In Scene 2 small incident angles), *H*(1 − *A*_12_) produced the best MC results in most cases. Generally, the features demonstrated better discrimination performance between two given targets for small incident angle than for large incident angles. The discrimination performance of some features was more adversely affected by the incident angle than others, indicating that the appropriate feature selection is highly important for a particular application.

## 4. Discussion

### 4.1. Analysis of Oil Slick Signal for Different Incident Angles

In this study, two images from the oil spill experiment in the Norwegian North Sea were selected for a comprehensive comparison under different incident angle conditions. The effect of the incident angle can be divided into three main aspects:

(1) The effect of noise is large at large incident angles; this increases the complexity and randomness of the target signal. In contrast, at small incident angles, the co-polarization channels are above the NESZ baseline, and only the cross-polarization channel is affected by noise.

(2) The incident angle affects the scattering mechanism. Li et al. [6] proposed that “When incidence angles are near 45°, especially at the grazing angle, Multipath Dihedral-Type Features are potentially important ocean surface scatterers.” Under such conditions (Scenes 1), the sea surface cannot be represented by a single type of Bragg scattering; instead, the superposition of multiple scattering mechanisms is used. This increases the randomness of the seawater signal, thus reducing the difference between the oil and seawater signals.

(3) The oil slick characteristics. This can be attributed to another possible effect, i.e., the time interval of 11 h between Scenes 1 and 2 [20]. In Scene 2 (small incident angles), the oil slicks were exposed to the marine environment for a longer time compared with those in Scene 1. In particular, the emulsified and plant oils may have undergone evaporation, emulsification, and diffusion, which may have changed the oil slick characteristics.

These factors affect the polarimetric feature expression of oil spill image inversion. Some studies have analyzed changes in oil spill characteristics and the differences in incident angles, which is beneficial for the quantitative assessment of the polarization characteristics of oil spills [20,24]. However, due to the combined effects, it is impossible to quantitatively determine the effect of a single change indicator. Therefore, research on the influence of a single variable indicator on polarimetric features under controlled conditions will help clarify the contribution of the differences in oil spill characteristics, and it is also key research in the future.

### 4.2. Advantages of Multitype Features for Different Types of Oil Slicks

The effective and promising polarimetric features are mainly manifested as follows: large inter-class differences and small intra-class dispersion. In particular, the dominant features should generate prominent oil slick signals while effectively suppressing sea clutter and false alarm information.

Among the selected polarimetric features, those related to the scattering mechanism achieved the best overall performance, followed by those related to channel correlation. However, slight differences were observed in the results, depending on the incident angle. Among the selected polarization features, the differences between target signals were generally smaller at large incident angles than at small incident angles. This can be attributed to the effect of the incident angle on the oil slick signal; however, the effect of time on the oil slick characteristics cannot be disregarded.

In addition, analyzing different types of features, the effectiveness of feature combinations was analyzed. The combined feature set *H_A*_12_ demonstrated a superior performance compared with its component features and the conventional combined feature set *H_A.* The feature combinations were obtained by applying mathematical operations to two features related to the scattering mechanism; consequently, they exhibited characteristics similar to those of the component polarimetric features. Additionally, the contrast between different targets was smaller at large than at small incident angles. Within the *H_A*_12_ feature set, *H*(1 − *A*_12_) and *A*_12_(1 − *H*) provided optimal results, further improving the application scenarios of the improved features. Therefore, at different incident angles, selecting the corresponding advantageous features for oil spill detection is conducive to constructing an advantageous feature set and assisting classification algorithms.

## 5. Conclusions

The unique and rare opportunity to conduct experiments on the spillage of different types of oil in the Norwegian Sea provided a special dataset for analyzing and comparing the characteristics of different types of oil slick under different time and incident angle conditions in the same oil spill experiment. We compared the relative distribution of signals and the noise baseline in the oil spill scenarios for different incident angles; additionally, we comprehensively analyzed the performance of multitype polarimetric features that were based on the scattering mechanism, channel correlation, and backscattering energy. Combined with qualitative (visualization of the PDF distribution) and quantitative (statistical features, MC measure, overlap ratio, and classification contribution) analyses, we comprehensively evaluated the detection performance of multitype features for different types of oil slicks. In addition, based on the performance characteristics of the combined feature sets in the two oil spill scenarios, we analyzed the differences in the dominant features for different incident angles. The polarimetric feature based on the scattering mechanism achieved the best overall performance, followed by that based on the channel correlation. Between different incident angle scenarios, the target feature differences in the small incident angle scenario are overall greater than those in the large incident angle scenario, especially in the detection of mineral oil and seawater. Among the combined *H*_*A*_12_ and *H_A* features, *H*(1 − *A*_12_) achieved the best performance at small incident angles; for large incident angles, *A*_12_ (1 − *H*) achieved the best performance, followed by *H*(1 − *A*_12_). These results indicate that selecting the corresponding advantageous features for different incident angles improves the detection performance for different oil types.

Furthermore, we comprehensively compared and analyzed the oil spill detection performance of different polarimetric features for different incident angles and demonstrated the potential of the combined feature set *H_A*_12_. Notably, this study was based on a limited dataset obtained from oil spill experiments. Tests using other datasets may produce different results owing to factors such as oil spill scenario, observation conditions, and sensor variations. In future research, we will consider the effects of different sensor attributes (e.g., spaceborne/airborne platform, mode, band, and NESZ baseline) and imaging conditions (e.g., incident angles, wind speed, wave height, seawater characteristics, and climate change). Furthermore, we will quantitatively compare and analyze the effect of changes in a single condition indicator on polarimetric features, such as changes in the incident angle and physical properties of oil spills, to meet the detection requirements for other types of oil spills. Finally, we will consider and compare the effects of additional parameters under different polarization modes (e.g., compact and dual polarization) to identify advantageous polarimetric features for oil spill detection under multisource conditions.

## Figures and Tables

**Table 1:** Parameters of RADARSAT-2 data used to characterize oil slick types.

Sensor	RADARSAT-2				
Frequency	C-band (5.405 GHz)				
Mode/Product	Fine Quad-Pol SLC				
Polarization	HH, HV, VH, VV				
Look Direction	Right				
Resolution (Rg × Az)	5.2 × 7.6 (m)				
Pixel space (Rg × Az)	4.7 × 4.8 (m)				
Region	The North Sea (59°59′ N, 2°27′ E)				
	Scene-1		Scene-2		
Scene/Date	8 June 2011 05.59		8 June 2011 17.27		
Incidence angle	46.1–47.3°		34.5–36.1°		
Wind speed/direction	1.6–3.3 m/s		1.6–3.3 m/s		
Slicks Information	Emulsion	plant	Crude	Emulsion	plant
Slicks present age	18 h	2 h	9 h	29 h	13 h

**Table 2:** Details of the representative polarimetric features used to identify marine oil spills.

Category	Description	Abbreviation/Reference/Definition	Oil Slick	Sea
I. Backscatter energy	Difference in the backscattering intensity between oil slick and sea	$\tau ={\langle \left|S_{HH}+S_{VV}\right|\rangle }^{2}/\sum_{i=1}^{i=3}\lambda_{i}$ [10]	Lower	Higher
		$PR={\langle \left|S_{HH}\right|\rangle }^{2}/{\langle \left|S_{VV}\right|\rangle }^{2}$ [19,20,24]	Higher	Lower
II. Scattering mechanism	Difference in the scattering mechanism between oil slick (non-Bragg) and seawater (Bragg)	$PH=\lambda_{3}/\lambda_{1}$ [5,25]	Higher	Lower
		$H=\sum_{i=1}^{3}-P_{i}\log_{3}P_{i}$ [19,26]	Higher	Lower
III. Channel correlation	Difference in the correlation between channels corresponding to an oil slick and the sea	$\rho \_\mathrm{co}=\frac{\left|\langle \left|S_{HH}S_{VV}^{*}\right|\rangle \right|}{\left|\sqrt{{\langle \left|S_{HH}\right|\rangle }^{2}{\langle \left|S_{VV}\right|\rangle }^{2}}\right|}$ [20,24]	Lower	Higher
		μ=2ReSHHSVV∗−SHV2SHH2+2SHV2+SVV2 [3]	Lower	Higher

**Table 3:** The Statistical indicator of signal-to-noise difference in targets in Scenes 1 and 2.

	Index	Signal-to-Noise Difference Indicators			
Class Label		Max	Min	Mean	Standard Deviation
		VV/HH	VV/HH	VV/HH	VV/HH
Scene 1	Emulsion	9.3/8.7	−7.1/−14.2	1.13/−1.5	3.53/3.91
	Plant	9.6/7.9	−6.1/−10.6	2.18/0.26	4.23/4.59
	Sea	18.07/13.5	−2.4/−7.5	9.05/3.24	3.99/4.09
Scene 2	Crude	8.74/8.2	2.1/1.3	5.5/4.62	1.34/1.48
	Emulsion	9.3/8.6	3.76/1.5	6.73/5.7	1.27/1.56
	Plant	15.6/13.7	6.6/4.3	10.43/8.91	1.76/1.83
	Sea	21.16/17.9	13.8/8.23	16.03/13.6	1.89/1.93

**Table 4:** MC results of multi-types polarimetric features in Scenes 1 and 2.

	Index	MC					
Class Label		*H*	*PH*	*µ*	*p_co_*	*PR*	*t*
Scene 1	Emulsion–Sea	0.2071	0.5183	0.1784	0.3114	0.2834	0.1701
	Plant–Sea	0.1697	0.379	0.1154	0.2173	0.1798	0.1124
	Emulsion–Plant	0.0388	0.1734	0.0643	0.101	0.1092	0.0588
Scene 2	Crude–Sea	0.5504	0.8476	0.2635	0.3754	0.1735	0.2162
	Emulsion–Sea	0.4956	0.7426	0.1843	0.273	0.1127	0.1546
	Plant–Sea	0.2757	0.3915	0.0437	0.0655	0.0915	0.0369
	Crude–Plant	0.3239	0.6825	0.2224	0.3177	0.0833	0.1807
	Emulsion–Plant	0.2548	0.495	0.1418	0.2112	0.0214	0.1183
	Crude–Emulsion	0.0754	0.2832	0.0832	0.1141	0.062	0.0637

**Table 5:** Statistical characteristics of the selected polarimetric features.

	Index	Mean/Standard Deviation						
Data		*H*	*PH*	*µ*	*p_co_*	*PR*	*t*	
Scene 1	Crude	0.8322	0.2283	0.5298	0.4123	0.8743	0.5875	$\bar{x}=\;\frac{\sum_{i=1}^{n}x_{i}}{n}$ *x*_i_: each data value *n*: sample number $\sigma =\;\sqrt{\frac{\sum_{i=1}^{n}{\left(x_{i}-\bar{x}\right)}^{2}}{n}}$*x*_i_: data value $\bar{x}$: mean value *n*: sample number
		**0.0536**	0.0567	0.0637	0.0677	0.1548	0.0556	
	Emulsion	0.7155	0.1276	0.6259	0.5186	0.7722	0.6675	
		0.0663	**0.032**	0.0633	0.0905	0.0977	0.0589	
	Plant	0.4249	0.0431	0.8327	0.7963	0.7399	0.8467	
		0.0652	**0.0119**	0.0352	0.0468	0.0961	0.0437	
	Sea	0.2413	0.0188	0.9087	0.9079	0.6158	0.9116	
		0.0353	**0.0042**	0.0147	0.0172	0.0584	0.0342	
Scene 2	Emulsion	0.8788	0.3306	0.543	0.3616	0.6328	0.5033	
		**0.0696**	0.104	0.0861	0.1023	0.1415	0.0745	
	Plant	0.8132	0.2329	0.6176	0.4428	0.5082	0.5662	
		0.0475	**0.0444**	0.0529	0.0726	0.0862	0.0476	
	Sea	0.5773	0.1049	0.7787	0.6886	0.3533	0.7097	
		0.0623	**0.0225**	0.0396	0.0519	0.0495	0.0412	

**Table 6:** ORs of multitype feature parameters.

	Index	Overlap (%)					
Class Label		*H*	*PH*	*µ*	*p_co_*	*PR*	*t*
Scene 1 40 × 40	Emulsion–Sea	**1.8125**	2.125	3.0625	2.50	9.12	2.81
	Plant–Sea	9.50	8.625	13.375	**8.38**	28.19	12.8125
	Emulsion–Plant	36.375	**31.75**	42.25	46.31	54.37	39.312
Scene 2 50 × 40	Crude–Sea	1.00	**0.95**	1.20	1.30	24.45	6.65
	Emulsion–Sea	4.90	4.90	**3.85**	4.100	32.65	11.50
	Plant–Sea	15.60	27.25	23.50	**13.00**	27.70	49.05
	Crude–Plant	7.35	**5.75**	7.50	9.20	57.25	11.90
	Emulsion–Plant	22.05	23.10	19.40	**19.35**	64.75	24.00
	Crude–Emulsion	68.75	**61.40**	71.75	82.25	87.25	74.00

**Table 7:** Classification accuracy of RF classifier.

		Class	Scene 1 (Large Incidence Angle)			Scene 2 (Small Incidence Angle)			
	Accuracy								
Features			Emulsion	Plant	Sea	Crude	Emulsion	Plant	Sea
I. Backscatter energy II. Scattering mechanism III. Channel correlation (*H PH µ pco PR t*)		Producer Accuracy (%)	82.39	88.87	99.41	86.55	72.22	82.16	98.67
		User’s Accuracy (%)	98.03	56.08	98.76	82.32	73.59	88.69	97.75
		Average Accuracy (%)	87.25			85.24			
		Kappa	0.8581			0.8242			
I. Scattering mechanism II. Channel correlation (*H PH µ pco*)		Producer Accuracy (%)	81.1	87.04	99.39	83.96	67.32	80.83	98.29
		User’s Accuracy (%)	97.45	53.83	98.74	79.61	70.26	85.4	97.22
		Average Accuracy (%)	86.25			82.861			
		Kappa	0.8463			0.7960			
I. Scattering mechanism (*H PH*)		Producer Accuracy (%)	80.44	86.81	99.27	82.51	62.74	72.63	97.87
		User’s Accuracy (%)	97.39	52.78	98.7	77.08	64.67	80.99	96.28
		Average Accuracy (%)	85.86			79.346			
		Kappa	0.8408			0.7561			

**Table 8:** Feature ratio (*R*_p_) and MC results of the combined feature *H*(1 − *A*_12_) and its sub-features for different targets in Scenes 1 and 2.

	Index	*H* (1 − *A*_12_)		*H*		1 − *A*_12_	
Class Label		*R*_p_	MC	*R*_p_	MC	*R*_p_	MC
Scene 1	Emulsion–Sea	3.6642	0.5712	1.5224	0.2071	2.4185	0.4149
	Plant–Sea	2.7255	0.4632	1.4086	0.1697	1.9571	0.3237
	Emulsion–Plant	1.3444	0.1469	1.0807	0.0388	1.2357	0.1054
Scene 2	Crude–Sea	22.4707	0.9148	3.4487	0.5504	6.6553	0.7387
	Emulsion–Sea	15.5432	0.8791	2.9652	0.4956	5.3102	0.6831
	Plant–Sea	4.0148	0.6012	1.7611	0.2757	2.2648	0.3874
	Crude–Plant	5.597	0.6968	1.9583	0.3239	2.9386	0.4922
	Emulsion–Plant	3.8715	0.5894	1.6837	0.2548	2.3446	0.402
	Crude–Emulsion	1.4457	0.1822	1.1631	0.0754	1.2533	0.1124

**Table 9:** MC results for *H_A* in the sample areas of Scenes 1 and 2.

	Index	MC							
Class Label		*HA*	*HA*_12_	*H*(1 − *A*)	*H*(1 − *A*_12_)	*A*(1 − *H*)	*A*_12_(1 − *H*)	(1 − *H*)(1 − *A*)	(1 − *H*)(1 − *A*_12_)
Scene 1	Emulsion–Sea	0.2022	0.1390	0.2081	0.5712	0.5515	**0.7067**	0.5550	0.2272
	Plant–Sea	0.2051	0.0323	0.1619	0.4632	0.3677	**0.5336**	0.3910	0.0788
	Emulsion–Plant	0.0031	0.1072	0.0479	0.1469	0.2306	**0.2778**	0.2094	0.1511
Scene 2	Crude–Sea	0.4197	0.2128	0.6135	**0.9148**	0.7252	0.8075	0.5804	0.1725
	Emulsion–Sea	0.491	0.2602	0.4989	**0.8791**	0.4609	0.6304	0.4501	0.3152
	Plant–Sea	0.2988	0.2111	0.2579	**0.6012**	0.1132	0.1983	0.1559	0.2552
	Crude–Plant	0.1383	0.0018	0.4224	0.6968	0.6667	**0.7253**	0.4667	0.0865
	Emulsion–Plant	0.2253	0.052	0.2765	**0.5894**	0.3669	0.4937	0.3163	0.0652
	Crude–Emulsion	0.0899	0.0502	0.1652	0.1822	**0.397**	0.3608	0.1764	0.1509

## Data Availability

The original contributions presented in this study are included in the article. Further inquiries can be directed to the corresponding author.

## Abbreviations

SAR	Synthetic aperture radar
FP	Fully polarimetric
NESZ	Noise equivalent sigma zero
NRCS	Normalized radar cross-section
MC	Michelson contrast
OR	Overlap Ratio
RF	Random forest
